# Perceived Exercise Benefits and Barriers of Non-Exercising Female University Students in the United Kingdom

**DOI:** 10.3390/ijerph7030784

**Published:** 2010-03-01

**Authors:** Geoff P Lovell, Walid El Ansari, John K Parker

**Affiliations:** 1 School of Social Sciences, Faculty of Arts and Social Sciences, University of the Sunshine Coast, Queensland, Australia; E-Mail: glovell@usc.edu.au; 2 Faculty of Sport, Health & Social Care, University of Gloucestershire, Gloucester, UK; E-Mail: jparker@glos.ac.uk

**Keywords:** physical activity, female university students, motivation, benefits, barriers, non-exercising

## Abstract

Many individuals do not engage in sufficient physical activity due to low perceived benefits and high perceived barriers to exercise. Given the increasing incidence of obesity and obesity related health disorders, this topic requires further exploration. We used the Exercise Benefits/Barriers Scale to assess perceived benefit and barrier intensities to exercise in 200 non-exercising female university students (mean age 19.3 years, SD = 1.06) in the UK. Although our participants were selected because they self reported themselves to be non-exercising, however they reported significantly higher perceived benefits from exercise than perceived barriers to exercise [*t*(199) = 6.18, *p* < 0.001], and their perceived benefit/barrier ratio was 1.33. The greatest perceived benefit from exercise was physical performance followed by the benefits of psychological outlook, preventive health, life enhancement, and then social interaction. Physical performance was rated significantly higher than all other benefits. Psychological outlook and preventive health were not rated significantly different, although both were significantly higher than life enhancement and social interaction. Life enhancement was also rated significantly higher than social interaction. The greatest perceived barrier to exercise was physical exertion, which was rated significantly higher than time expenditure, exercise milieu, and family discouragement barriers. Implications from this investigation for the design of physical activity programmes include the importance, for females, of a perception of high benefit/barrier ratio that could be conducive to participation in exercise. Applied interventions need to assist female students to ‘disengage’ from or overcome any perceived ‘unpleasantness’ of physical exertion during physical activity (decrease their perceived barriers), and to further highlight the multiple health and other benefits of regular exercising (increase their perceived benefits).

## 1. Introduction

The benefits of regular physical activity (PA) for physiological and psychological health are well documented [1]. However, despite the well publicised benefits of PA, many individuals from developed countries do not engage in PA sufficient for health benefits. For example, the 2008 survey of Queenslanders, Australia [2] showed that 53% of the adult population aged 18−75 years did not report PA levels sufficient for health benefits, with their median sitting time being 4.7 hours per day. Furthermore, approximately one in four adults (27.7%) were sedentary for an average of seven hours or more every day of the week [2]. In 2003, insufficient PA was the third largest single determinant of burden of disease in Queensland, associated with 6.2% of the burden for males and 6.8% for females [3]. Such findings are echoed in other high-income countries such as the UK and the USA. The Health Survey for England [4] reported that in 2006, only 40% of men and 28% of women met the American College of Sports Medicine (ASCM) guidelines for PA [5] (30 minutes of at least moderate-intensity activity on most days of the week). Likewise, in 2007, over 50% of American adults did not achieve the ACSM target level of PA for health benefits. Moreover, almost 25% reported no leisure time PA [6]. Insufficient PA and associated negative health outcomes are particular concerns for females, who at all ages are reported to be less physically active than males [7]. Women who exercise 3−4 hours per week are about 60% less likely to be obese compared to those who do not exercise [8]. Further, the benefits of exercise are accessible to the majority of the population, with reports suggesting that levels of PA that are attainable by ‘ordinary’ people are preventive for coronary heart disease [9].

Understanding why individuals do not participate in sufficient PA is complex and multifaceted-encompassing personal, interpersonal, environmental, and policy determinants. Research which advances our understanding of any of these factors has strong potential to better inform PA promotion interventions and thus support positive public health outcomes, both physiological and psychological. To date, the long term success of strategies to increase PA in adult women has been insufficient, and in order to develop effective health strategies, it is necessary to further investigate women’s motives for PA and the challenges they face in attempting to be active [10]. Within this context, the perceived benefits and barriers to exercise are important mediators of PA behaviour change [11]. Analysis of factors that influence women’s participation in PA has suggested that women who perceived more benefits from exercise and fewer barriers to exercise were typically more active than those who reported high perceived barriers and low perceived benefits [12]. These findings are consistent with the theoretical framework adopted by the present investigation, the health belief model (HBM) [13]. HBM, a cognitive behaviourist theory, contends that an individual’s readiness to engage in preventive health behaviour is a function of their perceived threat associated with that behaviour, e.g., physical inactivity, and an assessment of the relative costs (barriers, difficulties or hindering factors) and benefits associated with the adoption of that specific preventive health behaviour. The HBM’s original four constructs comprised: perceived susceptibility, perceived severity, perceived benefits, and perceived barriers. This proposes that the likelihood that an individual will engage in a health behaviour (e.g., PA) depends largely on their perceived magnitude of the barriers against being physically active, and their perceived benefits to being physically active. Although the literature has reported perceived barriers to be key in predicting health behaviour [13], El Ansari and Phillips’s more recent research has suggested the issue to be more complex, implicating the ratio of perceived barriers to perceived benefits as being more predictive of behaviour [14]. It needs also be considered, however, that psycho-social factors such as self-efficacy; demographic features such as age; personality and peer pressure; and other factors such as knowledge also play important roles in engagement and adherence to PA behaviour change interventions [15].

With specific reference to barriers, despite a recent review of about 50 studies of health behaviour change where perceived barriers were the single most powerful predictor of health behaviour [13], such barriers to exercise have not been examined in detail [16]. In addition, the limited number of studies that examined females’ perceived benefits and barriers to exercise have often not considered how different developmental milestones and specific circumstances at different stages of women’s lives impact on both their interest to exercise and ability to be physically active [17,18]. Furthermore, a characteristic of many developed countries is the relatively high percentages of the populous who enrol for higher education: e.g., in the USA during 2000 over 14.5 million students were enrolled in colleges and universities, with over 56% being women [19]. However, of the limited research that focused on university females, Irwin’s [20] review detailed a range of previous studies indicating that only between 28% and 50% of female university students engaged in sufficient PA, compared to between 40% and 68% of male students.

University students not only represent a specific under-researched population that in light of Irwin’s findings would benefit from increased PA levels, but university education contexts also provide “pivotal settings” of unrealized opportunities to influence PA behaviours of young adults [19] (p. 116). This is important given recent findings that almost one quarter of all students starting university gain a significant amount of weight during their first semester, a fact that supports the need of effective strategies to assist these young-adults starting university maintain a healthy body weight [21]. Facilitating and nurturing PA at university appears to have a similar role in shaping life-long PA behaviour as does schooling [22], with PA patterns possibly remaining stable for up to five years post graduation [23]. Hence the benefits of motivating university students to be active are twofold: (1) for direct PA behaviour outcomes that are associated with public health benefits; and, (2) for longer term outcomes.

Effective PA promotion programs and interventions require an evidence base of female students’ attitudes toward exercise in terms of perceived benefits and barriers. However, only few studies have examined female university students’ attitudes towards exercise [24]. Of the little research that considered female university students (e.g., [19,24]), non-exercising populations have not been examined. Such a gap in knowledge related to the perceived benefits and barriers to exercise for female students, specifically those who do not achieve recommended PA levels, hinders the development of successful population specific PA strategies targeting such females. The study described in this paper addresses this gap.

### 1.1. Aims of the Study

The purpose of this study was to examine the perceived exercise benefits and barriers of non-exercising female university students as defined by ACSM guidelines [25] and measured by the Exercise Benefits/Barriers Scale (EBBS) [26]. Findings from the current study should assist health and fitness practitioners, researchers as well as policy makers, to design more appropriate initiatives to better suit the individual needs of female university students in order to ultimately increase their PA levels. The specific objectives were to:

- Describe the sample’s general levels of perceived benefits and barriers to exercise;
- Assess whether non-exercising female university students had greater total perceived benefits or barriers to exercise;
- Identify what non-exercising female university students perceived to be the biggest benefits of exercise;
- Assess what non-exercising female university students perceived to be the biggest barriers to exercise; and,
- Identify how non-exercising female university students’ perceptions of benefits from exercise related to their perceptions of barriers to exercise.

## 2. Methods

### 2.1. Sample and Participants

Following university ethics approval, females volunteers (N = 200) were randomly selected from two universities in south England, UK. Females were eligible to participate in this study if they reported to be non-exercising and free from disease. “Non-exercising” was defined as an individual not meeting the ACSM recommendation to accumulate 30 minutes of moderately vigorous exercise on most days over a week [25]. Data collection was undertaken on three separate occasions at each university (Tuesday afternoon, a Wednesday morning, and a Thursday afternoon), mid way through the first semester of the 2008 academic year.

The survey data were collected by female students assisting in this research. It was anticipated that the use of female students to collect data from female student participants would help to reduce the likelihood of social desirability of the participants’ responses to the survey. The study approached every 10^th^ female that passed through the main corridor of the universities’ main campuses. Each potential participant that was approached was informed that as part of a research project associated with the University, females who did not partake in 30 minutes of moderate intensity exercise most days of the week were being invited to complete a short questionnaire. Potential respondents were informed about the aims of the survey, and that participation entailed answering some short questions about their thoughts towards benefits and barriers to exercise, as well as some demographic/background questions. Participants were asked if they met the inclusion criteria, if so they were then informed that participation in the study was voluntary, that all information was confidential, that no record of respondents’ names would be made, and that participants were free to withdraw at any point that they wished to do so. If in agreement, the participant then completed the survey. Roughly, 80% of the females approached agreed to participate in the study, and completed the EBBS individually at time of recruitment. Participants’ mean age was 19.03 years (SD = 1.06) possibly reflecting the nature of the universities where participants were recruited. Roughly 91% were single, 5% were married or lived with their partner, and 4% of the females had one or more children. About 60% of the women approached did not meet the ASCM criteria.

### 2.2. Instruments and Procedure

Perceived benefit and barrier intensities to exercise were assessed by the EBBS questionnaire [26] that comprised two components: Benefits and Barriers (Box 1). The benefit component comprised of 29 benefit items categorised into five subscales: life enhancement, physical performance, psychological outlook, social interaction, and preventative health. The barrier component included 14 barrier items categorised into four subscales: exercise milieu; time expenditure; physical exertion; and family discouragement. The reported internal consistency (alpha) for the benefits and the barriers scales were 0.95 and 0.86 respectively, while test re-test reliability was 0.89 and 0.77 respectively [24]. The internal consistencies of the benefits and barriers scale for this sample were 0.95 and 0.86 respectively.

Box 1. Scales and Sub-scales of the Exercise Benefits/Barriers Questionnaire.*

**Table:** 

**Perceived Benefits to Exercise** (29 items)	**Perceived Barriers to Exercise** (14 items)
Life enhancement (8 items)	Exercise milieu (6 items)
Physical performance (8 items)	Time expenditure (3 items)
Psychological outlook (6 items)	Physical exertion (3 items)
Social interaction (4 items)	Family discouragement (2 items)
Preventative health (3 items)	

* All items of both the benefit and barrier scales were scored on a Likert 4-point response format where 1 = ‘strongly disagree’; 2 = ‘disagree’; 3 = ‘agree’; and 4 = ‘strongly agree’.

### 2.3. Data Analyses

SPSS v16 was employed for the analysis. For each participant, standardized scores were computed for both the total benefits and total barriers scales, as well as for each sub-scale (total score for scale or sub-scale divided by number of items included in that scale or sub-scale). The purpose of this adjustment to the same 1 to 4 Likert scale was to allow direct comparisons between scales and sub-scales. Possible scores ranged from 1 to 4; 4 represented the highest perception of benefit and perception of barrier. Research objective one (to describe the sample’s general levels of perceived benefits and barriers to exercise) was achieved by computing the means of the individual EBBS items. Research objective two (whether non-exercising female university students had greater total perceived benefits or barriers to exercise) was assessed by a single paired samples *t*-test. The third and fourth research objectives (what non-exercising female university students’ perceived to be the biggest benefits and barriers of exercise) were assessed by multiple paired sample *t*-tests to identify any significant differences between subscales (10 comparisons for the benefits scale; 6 comparisons for the barriers scale). The Bonferroni method was used to correct critical *p* values (*p* < 0.005 for the benefits scale; *p* < 0.008 for the barriers scale) while maintaining an alpha of 5% to control against an inflated alpha and the increased possibility of type I errors due to these multiple comparisons. The fifth and final research objective (how non-exercising female university students’ perceptions of benefits from exercise related to their perceptions of barriers to exercise) was assessed by the calculation of correlations between each of the benefit sub-scales with each of the barrier subscales (20 correlations). Again to control against potential type I error due to multiple comparisons, the Bonferroni method was used to correct critical *p* values (*p* < 0.002) while maintaining an alpha of 5%.

## 3. Results

Table 1 depicts the sample’s means and standard deviations for each item of the benefits sub-scales. Generally, these university females either agreed or strongly agreed with most of the benefits under examination, reflecting that they felt that many of the statements actually represented benefits of regular exercising. However, for some benefit items, the sample exhibited somewhat neutral scores (e.g., ‘exercise helps me decrease fatigue’; ‘exercise improves the quality of my work’; and, items of the social interaction sub-scale); or scores that approached the “agree” option of the response scale (e.g., ‘my disposition is improved by exercise’; ‘exercising increases my mental alertness’; ‘exercise allows me to carry out normal activities without becoming tired’; ‘exercising makes me feel relaxed’; and, ‘I will live longer if I exercise’). Participants agreed the least with the item: ‘exercising increases my acceptance by others’; and agreed the most with: ‘exercising increases my level of physical fitness’.

Table 2 depicts the sample’s means and standard deviations for each item of the barriers sub-scales. Generally, participants fairly agreed with many of the barriers items, reflecting that they felt that several of the statements actually represented barriers to their regular PA. However, for some barrier items, there was clear-cut disagreement indicating that statements do not represent barriers (e.g., ‘exercise takes too much time from family relationships’; ‘my family members do not encourage me to exercise’). Participants’ disagreed most with: ‘I am too embarrassed to exercise’, while agreeing most with the items: ‘places for me to exercise are too far away’ and ‘exercise tires me’, closely followed by ‘exercise is hard work for me’.

Findings to the second research objective showed that this sample of non-exercising female university students felt significantly higher perceived benefits (M = 2.96, SD = 0.44) than barriers (M = 2.22, SD = 0.46) to exercise (*t*(199) = 6.18, *p* < 0.001). This equated to a benefit/barrier ratio of 1.33; the ratio being >1 demonstrated that these females perceived greater benefits than barriers (Table 3).

Regarding the study’s third objective, the greatest perceived benefit from exercise was physical performance (M = 3.25) followed by psychological outlook, preventive health, life enhancement, and social interaction (Table 3). The Table shows that physical performance was rated significantly higher (M = 3.25) than all other benefits. Respondents did not rate psychological outlook and preventive health significantly differently, although both were rated significantly higher than life enhancement and social interaction. Life enhancement was also rated significantly higher than social interaction. Only physical performance, psychological outlook, and preventive health demonstrated standardized means >3 which represented ‘true’ agreement that these statements comprised of factors that the sample viewed as benefits.

With reference to our fourth objective, the greatest perceived barrier to exercise was physical exertion followed by time expenditure, exercise milieu, and family discouragement. Physical exertion was rated significantly higher than all other barriers. There were no further significant differences between time expenditure, exercise milieu, or family discouragement (Table 3). Mean scores for all four barriers were between 2 and 3 which equated to between ‘agree’ and ‘disagree’ on the EBBS scoring scale, which we interpreted to mean neutral.

In connection with our fifth objective, the barrier of exercise milieu was significantly and negatively correlated with all five of the benefit sub-scales (Table 4). The barrier of family discouragement was correlated with all the benefit subscales with the exception of social interaction. The barrier sub-scale of time expenditure was significantly correlated with all the benefit subscales with the exception of preventive health. The barrier subscale of physical exertion was only significantly correlated with the benefit sub-scale of life enhancement. All the significant correlations were negative; higher perceived barriers were consistently associated with lower perceived benefits to various extents and *vice versa*.

## 4. Discussion

Adequate PA has a critical bearing on wellbeing and quality of life [27]. University contexts present key opportunities to promote PA behaviour in young adult populations e.g., female students. However, there is lack of information regarding attitudes toward exercise of female university students who do not achieve PA sufficient for health benefits. This restricts the design of effective and specialized PA promotion programmes. The present study examined the perceived exercise benefit and barrier intensities of non-exercising female university students in the UK.

In connection with the first objective, the sample’s general levels of perceived benefits or barriers to exercise generally indicated that participants either ‘agreed’ or almost ‘strongly agreed’ with most of the benefits items, while only being neutral or at best approaching agreement with many of the barriers items. This suggested that our sample of university students perceived higher levels of benefits from exercise than barriers to exercise, and indeed their perceived benefit/barrier ratio was 1.33. For the benefits, participants agreed the least with ‘exercising increases my acceptance by others’, while agreeing the most with ‘exercising increases my level of physical fitness’. For the barriers, participants agreed the most with ‘places for me to exercise are too far away’, ‘exercise tires me’, closely followed by ‘exercise is hard work for me’; conversely, the strongest disagreement was with the barriers ‘exercise takes too much time from family relationships’, ‘my family members do not encourage me to exercise’, and ‘I am too embarrassed to exercise’.

As regards the second objective of this study, despite all our participants being classified as ‘non-exercising’ using ASCM guidelines [25], their perceived benefits were significantly greater than the perceived barriers to exercise. This is consistent with previous suggestions that perceived barriers could be more influential on behaviour than perceived benefits [11].

In relation to our third objective, for these non-exercising female university students, the strongest perceived benefit from exercising was physical performance. This was followed by psychological outlook and preventive health while life enhancement and social interaction benefits were notably lower. The finding that physical performance (encompassing multiple health aspects e.g., fitness, stamina, muscle tone, and physical appearance) was the highest perceived benefit from exercise, may not be surprising as the importance of such qualities for females are continually emphasized by a wide range of media channels. Similarly, the rating of psychological outlook as the second highest benefit from exercising is in support of Biddle and Bailey [28] who found that females were particularly appreciative of the enhancements in mental well being that resulted from exercise. It was also encouraging that our sample expressed a strong perception that exercise does provide positive preventive health benefits. Our finding that female university students were aware that exercise can aid their long term health is important in relation to the health belief model [13], as such perceptions could act as precursors to behavioural change. It is also reassuring that recent UK policy directives (e.g., two Government white papers: *Choosing health: Making healthier choices easier* [29], and *Choosing activity: A physical action plan* [30]), as well as advertising and school-based education programs appear to have been successful in alerting young adult females to the important health benefits associated with PA. Nevertheless, although this sample appeared to be aware of and moreover, valued the benefits of exercising, the fact that the females in this study were inactive suggested they were still not responding positively, or were yet to respond to such health education messages and information.

Our samples’ perception that there were relatively fewer benefits from exercising associated with life enhancement and social interaction factors is in contrast with some earlier literature, yet still plausible. Classically, previous research (e.g., [31]) and motivation theory (e.g., cognitive evaluation theory [32]) have suggested that social issues could be key motives for the continued participation in exercise programs. However, our sample represents a specific population (university students), often different to those participating in previous exercise behaviour and motivation studies. Hence the relatively fewer benefits from exercising associated with life enhancement and social interaction factors might be explained by that university students generally encounter numerous opportunities to meet people, socialize, mingle and interact – these are all an integral part of university life and college experience for these young adults. These abundant socialisation opportunities could have perhaps ‘undermined’ the perceived importance of the social benefits that could accrue from exercise.

In terms of the fourth objective, non-exercising female university students felt that family discouragement was the least barrier to exercise. This finding might be expected, as 91% of the sample was single. It was also encouraging to find that exercise milieu was not considered to present a meaningful barrier to exercise. This positive finding contrasts with King *et al.*’s [33] suggestion that young adult females find it difficult to exercise due to limited access to facilities. Furthermore, these results challenge the traditional views that females perceive exercising situations embarrassing or intimidating [24,34]. However, our findings might be specific to university students who are usually confident in their social contexts and with relatively open (and often free of charge) access to exercise facilities and PA opportunities.

Time expenditure was considered to be more of a barrier than both family discouragement and exercise milieu, although significantly less than physical exertion. The limited perception of time expenditure as a barrier to exercise is positive as it reflects potential time to exercise. The participants’ perceptions that the availability of time was rated ‘neutral’ as a barrier to exercise may reflect effective time management skills of these females, potentially developed through university education or possibly well-scheduled university exercise classes. Although our findings suggested that time was seen to be neutral as a barrier, time was still viewed to be a larger barrier than the exercise milieu. This is in agreement with Gyurcsik *et al.* who examined the barriers to PA in 198 Canadian students [24]. Gyurcsik found that 52% of their university students cited social invitations during workout time to be a barrier to PA and 74% cited their workload too high to allow for PA; both these aspects represent time expenditure barriers to some extent. Gyurcsik also found that exercise milieu issues were generally cited by fewer students, with only 3% cited lack of money as a barrier, although 62% cited transport as a barrier [24].

Physical exertion was significantly the largest perceived barrier to exercise. Our sample’s perception that the major barrier to exercise was that PA is fatiguing and hard work is of great concern. A vicious circle could be initiated: as students lose (regress) in their physical fitness condition, they could perceive that subsequent PA will usually be even harder. This in turn reinforces physical exertion as a barrier to exercise thus reducing their activity and in turn their physical fitness condition. The perception of physical exertion as the major barrier to exercise may also reflect a cultural or social phenomenon. According to Ajzen and Madden (theory of planned behaviour) [35], attitudes are affected by social norms, which then influence intentions and in turn behaviour. If the social norm is not to be physically active and not to enjoy the physical concomitance of being physically active (e.g., increased heart rate, increased sweating, feelings of being activated), then an individual’s attitude towards PA may become more negative, with the knock on effect of reduced exercise intention and ultimately behaviour. In the light of peer pressure, and current trends and social contexts of students, universities (and schools) that do not sufficiently project positive images about the health benefits of physical exertion might instead become effective environments for the propagation of negative perspectives towards physical exertion.

With regard to the fifth objective, many of the barrier subscales were significantly and negatively associated with individual benefit subscales. The barrier of exercise milieu was negatively associated with all the benefit sub-scales, although physical exertion was only associated with social interaction. The interrelation of some, but not of all the barrier and benefit subscales demonstrated the complexity of the nature of these factors. Furthermore, these linkages show how interventions focusing on different barriers could also have a potential positive effect on related perceived benefits, e.g., the linkage between exercise milieu and social interaction. These interconnections may also suggest indirect avenues to influence perceived barriers through planned management of females’ perceived benefits to PA.

The study has limitations. Findings of cross sectional studies are associations and do not infer causality. The sample comprised 200 female students representing a narrow age range, therefore caution needs to be exercised when attempting to generalize to other contexts or populations. Nevertheless, the data were collected via random selection at two universities, on three different occasions, thus increasing the potential to generalize of our findings to similar populations. As the data collected is self-reported, it was essential to minimise respondent burden, so by keeping the questionnaire short, no information was collected on women’s ethnicity, year of study, family care responsibilities, wider socioeconomic characteristics, or other possibly confounding variables. Further research would need to provide insights into how these different benefits/barriers factors function in respect to each other and/or as moderating variables. Longitudinal studies could also provide evidence on directions of causality.

## 5. Conclusions

Non-exercising female university students felt strong benefits from exercising accompanied by only relatively less barriers. It is possible that their perceived benefit/barrier ratio of 1.33 might not be sufficient to motivate these females to be physically active. University-based health education and PA promotion initiatives might encounter more effectiveness if such efforts focussed on educating non-exercising females as to how they could perceive a high (big) benefit/barrier ratio that would stimulate them to maintaining a physically active life style that benefits health. For instance, in the context of participation in health partnerships in South Africa, El Ansari and Phillips [14] showed that people will keenly participate in programmes and interventions if they perceive their accrued benefits from such participation to be *much more* than their difficulties (barriers). Active involvement and engagement were associated with a benefits/difficulties (barriers) ratio of about 80% more benefits than the difficulties of participation [14]. Perhaps such a similarly high ratio might be required for exercising in order to initiate and maintain females’ regular participation in PA programmes.

Other explanations could be that modern culture is applying pressure upon females to conform to social norms in terms of appearance, that females have become sensitized to any source of information, potentially including ill-informed popular media. Although such hypotheses may explain the high perceived benefits from exercising, such models do not explain the limited PA despite the small reported barriers to exercise. It could also be that females could be held in some form of chronic contemplative state of change [36,37], or that current social and cultural issues do not encourage nor support engagement in PA.

Implications of this study include the importance of applied interventions which consider a two-pronged approach. Interventions could help decrease the perceived barriers by ‘distracting’ or ‘disengaging’ female students from any perceived ‘unpleasantness’ of physical exertion during PA (e.g., by the use of cognitive strategies or music to re-direct the females’ attention away from the internal physiological cues associated with physical exertion). In addition, interventions could also further highlight the benefits and emphasize the paybacks of regular exercising to such populations in order to attract females to the various advantages and returns of PA. Interventions servicing one or (preferably) both of these two directions could increase the likelihood of engagement in exercise. Additionally, the findings support the proposal that age and context-specific participant groups must be utilised when attempting to gain meaningful insights into non-exercising females’ attitudes toward exercise and PA.

## Figures and Tables

**Table 1.** The exercise benefits scale: mean and standard deviation of each questionnaire item.*

**Perceived Benefit Items**	**M (SD)**
**Life Enhancement Sub-scale**	

25: My disposition is improved by exercise	2.94 (0.85)
26: Exercising helps me sleep better at night	3.14 (0.67)
29: Exercise helps me decrease fatigue	2.66 (0.65)
32: Exercising improves my self-concept	3.02 (0.72)
34: Exercising increases my mental alertness	2.90 (0.67)
35: Exercise allows me to carry out normal activities without becoming tired	2.93 (0.65)
36: Exercise improves the quality of my work	2.75 (0.73)
41: Exercise improves overall body functioning for me	3.08 (0.60)

**Physical performance Sub-scale**	

7: Exercise increases my muscle strength	3.20 (0.65)
15: Exercising increases my level of physical fitness	3.45 (0.66)
17: My muscle tone is improved with exercise.	3.25 (0.66)
18: Exercising improves functioning of my cardiovascular system	3.32 (0.62)
22: Exercise increases my stamina	3.14 (0.57)
23: Exercise improves my flexibility	3.11 (0.60)
31: My physical endurance is improved by exercising	3.18 (0.59)
43: Exercise improves the way my body looks	3.34 (0.65)

**Psychological Outlook Sub-scale**	

1: I enjoy exercise	3.05 (0.81)
2: Exercise decreases feelings of stress and tension for me	3.11 (0.80)
3: Exercise improves my mental health	3.03 (0.72)
8: Exercise gives me a sense of personal accomplishment	3.33 (0.72)
10: Exercising makes me feel relaxed	2.86 (0.72)
20: I have improved feelings of well being from exercise	3.13 (0.66)

**Social Interaction Sub-scale**	

11: Exercising lets me have contact with friends and persons I enjoy	2.61 (0.92)
30: Exercising is a good way for me to meet new people	2.56 (0.88)
38: Exercise is good entertainment for me	2.64 (0.77)
39: Exercising increases my acceptance by others	2.18 (0.76)

**Preventive Health Sub-scale**	

5: I will prevent heart attacks by exercising	3.12 (0.68)
13: Exercising will keep me from having high blood pressure	3.07 (0.61)
27: I will live longer if I exercise	2.97 (0.73)

**All Benefit items of all subscales**	**2.96 (0.44)**

* Adapted from the Exercise Benefits/Barriers Scale (EBBS) [26].

**Table 2.** The exercise barriers scale: mean and standard deviation of each questionnaire item*.

**Perceived Barriers Items**	**M (SD)**
**Exercise Milieu Sub-scale**	

9: Places for me to exercise are too far away	2.69 (0.70)
12: I am too embarrassed to exercise	1.85 (0.83)
14: It costs too much money to exercise	2.26 (0.86)
16: Exercise facilities do not have convenient schedules for me	2.09 (0.74)
28: I think people in exercise clothes look funny	2.04 (0.88)
42: There are too few places for me to exercise	2.10 (0.77)

**Time Expenditure Sub-scale**	

4: Exercising takes too much of my time	2.31 (0.81)
24: Exercise takes too much time from family relationships	1.95 (0.67)
37: Exercise takes too much time from my family responsibilities	2.04 (0.71)

**Physical Exertion Sub-scale**	

6: Exercise tires me	2.69 (0.70)
19: I am fatigued by exercise	2.57 (0.75)
40: Exercise is hard work for me	2.63 (0.70)

**Family Discouragement Sub-scale**	

21: My spouse (or significant other) does not encourage exercising	2.15 (0.87)
33: My family members do not encourage me to exercise	1.96 (0.65)

**All Barriers items of all subscales**	**2.22 (0.46)**

* Adapted from the Exercise Benefits/Barriers Scale (EBBS) [24].

**Table 3.** Standardized perceived benefit and barrier sub-scale means and standard deviations and *t*-test values for multiple comparisons.

**Sub-scale**	**Mean (SD)**	**Sub-scale†**				
		**1**	**2**	**3**	**4**	**5**
Benefits (M = 2.96, SD = 0.44)						

1. Physical performance	3.25 (0.46)	- -	6.36*	5.80*	11.80*	17.93*
2. Psychological outlook	3.08 (0.60)		- -	0.72	5.36*	14.22*
3. Preventive health	3.05 (0.56)			- -	3.57*	10.83*
4. Life enhancement	2.93 (0.48)				- -	11.97*
5. Social interaction	2.50 (0.65)					- -

Barriers (M = 2.22, SD = 0.46)						

1. Physical exertion	2.63 (0.60)	- -	11.37*	12.72*	10.27*	
2. Time expenditure	2.12 (0.59)		- -	1.39	1.35	
3. Exercise milieu	2.08 (0.60)			- -	0.39	
4. Family discouragement	2.06 (0.62)				- -	

For all subscales, possible scores range from 1 to 4, where 4 represents the highest perception of both benefits and barriers;

† Values in the cells of these columns are actual *t*-test values;

* Indicates that the means of the subscales that are being compared were significantly different, using Bonferroni corrected critical *p* values for benefits (*p* < 0.005) and for barriers (*p* < 0.008).

**Table 4.** Correlation coefficients between perceived barriers and benefits of exercise subscales.

**Benefit Sub-scale**	**Barrier Sub-scale**			
	Physical Exertion	Time Expenditure	Exercise Milieu	Family Discouragement
Physical performance	−0.030	−0.349*	−0.358*	−0.345*
Psychological outlook	−0.199	−0.418*	−0.466*	−0.312*
Preventative health	−0.100	−0.202	−0.316*	−0.345*
Life enhancement	−0.404*	−0.481*	−0.352*	−0.250*
Social interaction	−0.171	−0.237*	−0.352*	−0.198

* Significant correlations, using Bonferroni corrected critical *p* value (*p* < 0.002).
